# Supplementary material for: A specific role for serotonin in overcoming effort cost
Source: eLife. 2016 Nov 8;5:e17282. doi: 10.7554/eLife.17282 (PMC5100997; doi:10.7554/eLife.17282)
Supplement: Reporting standards 2. — DOI: http://dx.doi.org/10.7554/eLife.17282.013 [file elife-17282-repstand2.doc]

**
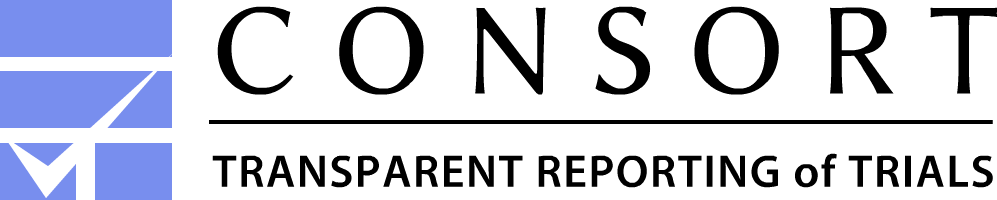
**

**CONSORT 2010 Flow Diagram**

**Allocation**

**Analysis**

**Follow-Up**

**Enrollment**

Assessed for eligibility (n=? )

Excluded (n= )

♦  Not meeting inclusion criteria (n= )

♦  Declined to participate (n= )

♦  Other reasons (n= )

Analysed (n = 29 subjects in total, but the exact number depends on the time since treatment onset. See table 1 for details. )
♦ Excluded from analysis (give reasons) (The number of excluded data set for each time since treatement onset is shown in table 1). See methods for criteria.

Lost to follow-up (give reasons) (n=0 )

Discontinued intervention (give reasons) (n=0 )

ESCITALOPRAM GROUP

Allocated to intervention (n=32 )

♦ Received allocated intervention (n=32 )

♦ Did not receive allocated intervention (give reasons) (n=0 )

Lost to follow-up (give reasons) (n=0 )

Discontinued intervention (give reasons) (n= 0 )

PLACEBO GROUP

Allocated to intervention (n= 32 )

♦ Received allocated intervention (n= 32 )

♦ Did not receive allocated intervention (give reasons) (n= 0 )

Analysed (n = 29 subjects in total, but the exact number depends on the time since treatment onset. See table 1 for details. )
♦ Excluded from analysis (give reasons) (The number of excluded data set for each time since treatement onset is shown in table 1). See methods for criteria.

Randomized (n=64 )
